# HaloTag-based reporters for sparse labeling and cell tracking

**DOI:** 10.1080/19336934.2022.2142460

**Published:** 2022-11-02

**Authors:** Lydie Couturier, Juan Luna, Khalil Mazouni, Claire Mestdagh, Minh-Son Phan, Francis Corson, Francois Schweisguth

**Affiliations:** a 4D Unit, Developmental and Stem Cell Biology Dept, Institut Pasteur, CNRS UMR3738, 75015 Paris, France; b Laboratoire de Physique de l’Ecole Normale Supérieure, CNRS, Sorbonne Université, Université Paris Diderot, 75005, Paris, France

**Keywords:** Cell tracking, single cell analysis, HaloTag, sparse labelling, cell shape, cell fate, live imaging, imaginal disc, *Drosophil*a, morphogenesis

## Abstract

Multiscale analysis of morphogenesis requires to follow and measure in real-time the *in vivo* behaviour of large numbers of individual cells over long period of time. Despite recent progress, the large-scale automated tracking of cells in developing embryos and tissues remains a challenge. Here we describe a genetic tool for the random and sparse labelling of individual cells in developing *Drosophila* tissues. This tool is based on the conditional expression of a nuclear HaloTag protein that can be fluorescently labelled upon the irreversible binding of a cell permeable synthetic ligand. While the slow maturation of genetically encoded fluorescent renders the tracking of individual cells difficult in rapidly dividing tissues, nuclear HaloTag proteins allowed for rapid labelling of individual cells in cultured imaginal discs. To study cell shape changes, we also produced an HaloTag version of the actin-bound protein LifeAct. Since sparse labelling facilitates cell tracking, nuclear HaloTag reporters will be useful for the single-cell analysis of fate dynamics in *Drosophila* tissues cultured *ex vivo.*

## Introduction

How ensembles of individual cells build and pattern complex tissues and multicellular organisms is not well understood. Insights into how progenitor cells contribute to the spatial organization of tissues and embryos have come from lineage tracing whereby individual cells are labelled with a genetically heritable marker that is detected in progeny cells after a certain period of time [1,2]. Lineage tracing, however, provides no information on the real-time developmental trajectory of individual cells. By contrast, cell tracking can give access to the behaviour of individual cells as they form complex structures. Recently, spectacular progress in microscopy and image analysis have paved the way for the cell tracking of whole embryos [3,4]. Indeed, automated cell tracking of cells can map in real time the behaviour of all cells in embryos, tissues and organoids. It can also produce atlases of embryos at single cell resolution with complete lineage trees and detailed description of cellular movements. However, large-scale automated cell tracking remains quite challenging. Indeed, correct and efficient automated tracking requires near-perfect segmentation together with short time intervals between acquisition time frames, which in turn implies imaging at high spatial and temporal resolution while minimizing light power to limit bleaching and phototoxicity. Since tracking hundreds or thousands of cells over several hours or days remains a formidable task, simpler methods would be useful to routinely study cell dynamics.

One approach to simplify the analysis of single cells over time is to follow only a subset of cells using random and sparse labelling of cells. This is because spare labelling dramatically facilitates the segmentation and tracking of the labelled cells and this approach has been extremely useful to study cell behaviour over time in various model organisms [5]. Despite this, sparse labelling has rarely been used in *Drosophila*. In this model organism, the usual approach to randomly label individual cells involves recombination whereby a transcriptional stop cassette is removed by FLP-FRT recombination to direct the expression of a fluorescent marker [6,7]. This approach culminated with the development of Flybow, a genetic multicolour tool that relies on the stochastic expression of different fluorescent proteins following recombination [8]. While other recombination strategies have also been used [9], none appears to be suited to rapidly label individual cells. Indeed, the time delays associated with recombination, gene expression, translation and maturation of the fluorescent proteins may be longer than the cell cycle measured in rapidly dividing fly tissues, making it challenging to label single cells. Moreover, given the limited cell mixing seen in fly tissues, the few marked cells produced by the division of the recombined cells are not sparse. Additionally, to study fate dynamics at the single cell level, it would be desirable to combine activity reporters with sparse labelling. Since most reporters for signalling and fate dynamics rely on GFP tagging, it would be optimal to use a spectrally distinct protein. Here, we address this issue by generating HaloTag-based reporters.

## Results and discussion

To circumvent the slow maturation times of all non-GFP fluorescent proteins, we used a genetically encoded self-labelling enzyme as a marker for cell tracking. The HaloTag protein is a modified haloalkane dehalogenase that was designed to covalently and irreversibly bind a small, cell permeable synthetic ligand such that it can be fluorescently labelled with high brightness and photostability [10]. It can be easily fused to a protein of interest and the labelling reaction of the HaloTag7 version is primarily limited by the diffusion of cell-permeant ligands [11]. HaloTag labelling is thus expected to be faster than the maturation times of red and infra-red fluorescent proteins which are in the 40–100 min range [12–14]. The HaloTag7 ligands have high signal-to-noise ratio and are commercially available with dyes that are excitable at 549 and 646 nm [15]. In addition, the labelled HaloTag proteins should produce a stronger signal that is more stable over time than any of the red and infra-red fluorescent proteins. These properties make this marker suitable for long-term imaging. However, the HaloTag ligands need to be provided directly to the cells and tissues, implying that cell tracking with HaloTag is best suited for cultured explants of developing fly tissues.

**Figure 1.**
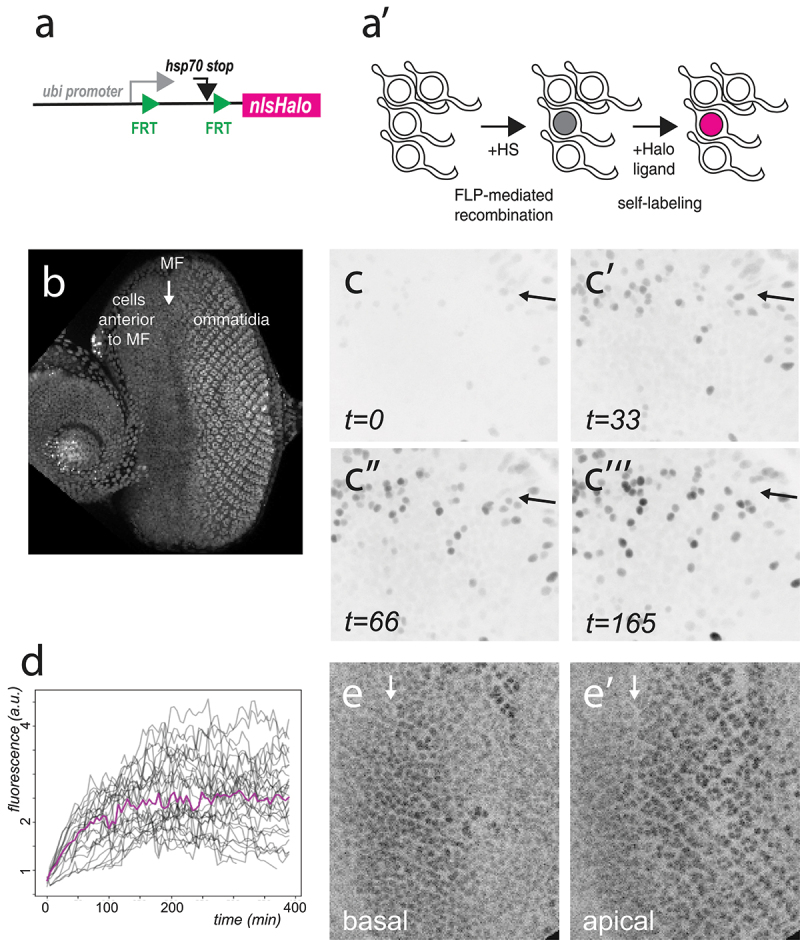
Cell tracking based on sparse nlsHalo labelling in eye imaginal discs. (a, a’) Structure of the pUbi-FRT-nlsHalo transgene prior to FLP-out excision (a) and cartoon illustrating the labelling of a single cell with nlsHalo (nucleus in magenta) upon low-frequency FLP-mediated recombination (a’). Detection of nlsHalo involves a time delay integrating time delays for FLP-mediated recombination (upon heat-shock, noted +HS) and synthesis of non-fluorescent nlsHalo protein in the living organism (nucleus in grey) and self-labelling with an Halo ligand in dissected tissue cultured and labelled *ex vivo* (nucleus in magenta). (b) Eye-antenna imaginal disc dissected from a third instar larva and cultured *ex vivo*. Nuclei were detected using the nlsRFP fluorescence signal. The position of the MF is indicated with an arrow. Anterior is to the left and ommatidia are posterior to the MF. (c-c”’) Snapshots of a sparse labelling movie showing randomly labelled nuclei expressing nlsHalo at the level of the MF (arrow). Time (t) is in minutes (min; the HaloTag ligand was added ~30 min prior to *t = 0*). Anterior is to the top. (d) Plot showing the level of the nlsHalo signal measured in single nuclei that were tracked over time from the movie shown in (c-c”’). Intensity values for individual tracked nuclei are shown in grey. Shown in magenta are mean intensity values of all nuclei, tracked and non-tracked. Time is in minutes (min). Imaging started at t = 0, while self-labelling, i.e. addition of the HaloTag ligand, started ~30 min prior to imaging. Sparse labelling facilitated the tracking of these cells. (e, e’) Snapshot views (e, apical and e’, basal) of a movie with all nuclei marked by nlsHalo. The position of the MF is indicated by an arrow. Anterior is to the left.

First, we generated a FLP-out transgene that directs the conditional expression of a nuclear HaloTag7 protein, nlsHalo, under the control of the strong *ubi* promoter (Figure 1a, a’). Constitutive expression of HaloTag7 was induced upon FLP-mediated excision of a transcriptional stop flanked by two FRT sites. To perform cell tracking, we used cultured eye imaginal disc explants (Figure 1b). The adult fly eye develops from a neuro-epithelium that progressively differentiates during the late third instar as a differentiation front, known as the morphogenetic furrow (MF), sweeps through the eye epithelium from posterior to anterior such that cells anterior to the MF are proliferative and non-differentiated whereas cells posterior to the MF are differentiated, mostly post-mitotic and adopt characteristic cell shapes to form a highly ordered epithelium [16]. Upon heat-induced expression of the FLP, stochastic recombination of the FRT stop resulted in the expression of the nlsHalo protein. We found that a brief and moderate heat-shock, i.e. 10–15 min at 32–34°C, was sufficient to induce recombination in 10–30% of the cells. Recombined cells were easily detected 14–16 hours at 18°C after heat-shock (equivalent to 7–8 hours at 25°C) and ~45-60 min after the addition of the HaloTag ligand (Figure 1c, c’), with labelling intensity reaching a plateau 2–3 hours later (Figure 1c”, c”’). Under our culturing conditions, cell tracking could be performed over 6–8 hours of imaging, a time scale that was sufficient to study fate dynamics in the eye imaging disks (Figure 1d). While sparse labelling facilitates tracking irrespective of a particular image analysis method, we note that this labelling strategy should be particularly useful in tissues where cells, or nuclei, are densely packed and/or move around rapidly. In this context, our genetic tool has the advantage that the density of labelled nuclei, i.e. the frequency of recombination, can be easily tuned through optimization of the heat-shock conditions. We conclude that HaloTag labelling can facilitate cell tracking for the study of morphogenesis of *Drosophila* tissues cultured *ex vivo*.

We next generated a line expressing constitutively nlsHalo by excising the FRT-stop cassette in the germ-line. This produced a bright nuclear marker that can be labelled in the infra-red (Figure 1e, e’). This labelling strategy provides an excellent signal-to-noise ratio upon low excitation light as well as stable brightness over time, hence facilitating segmentation. Additionally, infra-red labelling is useful for three-colour imaging when using GFP- and RFP-based markers.

**Figure 2.**
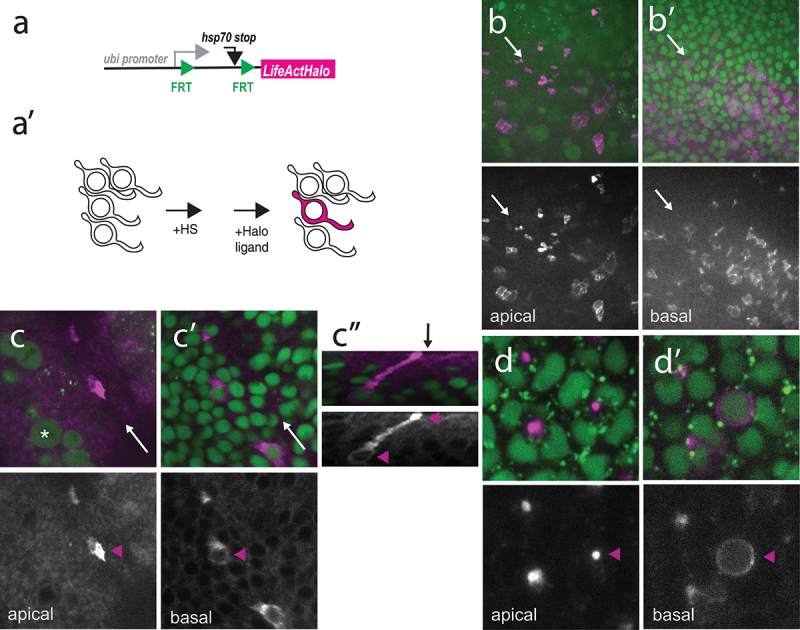
Cell shape analysis using sparse LifeActHalo labelling in eye imaginal discs. (a, a’) Structure of the pUbi-FRT-LifeActHalo transgene prior to FLP-out excision (a) and cartoon showing the labelling of a single cell with LifeActHalo (cellular cortex in magenta) upon heat-induced recombination and self-labelling (see Fig 1A’). (b, b’) Snapshot of an eye disc cultured ex vivo showing randomly labelled cells expressing LifeActHalo (magenta) at the level of the MF (arrow). In apical sections, MF cells appeared apically constricted (b). In contrast, labelled cells located anterior to the MF formed small clusters of 2–4 cells. Cortical staining was observed at the apical surface (b) and more basally at the level of the nuclei (b), marked with H2Av-mRFP1 (green). Anterior is to the bottom left. (c-c”) Snapshot of a pair of MF cells labelled with LifeActHalo (magenta). Nuclei were detected using H2Av-mRFP1 (green). The position of the MF is indicated with arrows (white in c, c’, black in c”). Anterior is to the left. The MF cells expressing LifeActHalo has a constricted actin-rich apex (c; magenta arrowhead in the bottom panel) and basal nuclei (c’; magenta arrowhead in the bottom panel; one nucleus is detected at this z-position). Several cells anterior to the furrow divide apically (asterisk). An orthogonal reconstructed section of this cell pair is shown in c” (the z-positions of the sections shown in c, c’ are indicated with magenta arrowheads; apical, top). Note the apical enrichment of F-actin. Also, only one of the two basal nuclei is observed in this reconstructed section. (d, d’) Snapshot of randomly labelled differentiated cells (LifeActHalo, magenta). Apical (d) and basal views (d’) are shown. A single photoreceptor cell is indicated with arrowheads (magenta, bottom panels). Nuclei were detected using H2Av-mRFP1 (green).

Last, we also generated a sparse labelling reporter directing the expression of an HaloTag protein fused to a short F-actin binding peptide known as LifeAct (Figure 2a). This LifeActHalo protein marks the cellular cortex and reveals the shape of individual cells following labelling (Figure 2a’). Using mild heat-shock, the random labelling of eye disk cells with LifeAtcHalo was observed 14–16 hours at 18°C after FLP-induction (Figure 2b, b’). Pairs of cells were most often detected anterior to and within the MF (Figure 2b-c”) and individually labelled MF cell were rarely detected. This indicated that many of the non-differentiated cells that underwent recombination also divided in the time period before imaging (see dividing cells anterior to the MF in Figure 2c). In contrast, single ommatidial cells were routinely detected posterior to the MF (Figure 2d, d’), presumably because differentiated eye cells are post-mitotic. Our attempts to more efficiently produce single cell clones in the MF were unsuccessful. Indeed, reducing the time interval between heat-shock, i.e. FLP-out recombination, and self-labelling of the Halo marker resulted in lower levels of the HaloTag signal but did not increase the proportion of individual cells in the MF (not shown). One possible interpretation is that addition of Insulin in the culturing medium induced rapid cell division, hence 2-cell clones. Thus, finding an optimal compromise between sensitivity and stochasticity will depend on various biological and technical detection parameters. In summary, this marker should in principle be useful to monitor the dynamics of cell shape changes and correlate fate with shape in single cells during morphogenesis to provide a temporal dimension to single-cell morphometrics [17] but achieving this in the eye disc may require changes in culturing conditions.

In conclusion, we have developed a new genetic tool for the automated tracking of individual nuclei in developing imaginal discs cultured *ex vivo*. This tool will be useful to combine cell tracking with the real-time quantitative analysis of GFP reporters and/or endogenous GFP-tagged proteins. One limitation of HaloTag proteins, however, is that labelling is most easily performed *ex vivo*. Nevertheless, *in vivo* cell labelling might be performed in embryos, larvae and pupae following the injection of HaloTag ligands. Future experiments will address the feasibility of injection approaches for cell tracking *in vivo*.

## Materials and methods

### Transgenes and flies

The pUbi-FRT-nlsHalo plasmid was obtained by modifying the psqh-Gap43-mCherry plasmid (kindly provided by A. Martin, MIT) in several steps by Gibson assembly. Briefly, the sequence of the *hsp70* transcriptional stop flanked by FRT sites (kindly provided by R. Holmgren, Northwestern U.) was inserted within the 5′UTR of the *sqh* gene, the *sqh* promoter was replaced by the 1986 nt-long region upstream of the ATG of the *p63E* gene, and the Gap43-mCherry fusion was replaced by nls-Halo7 or LifeAct-Halo7. The Halo7 sequence was obtained by genomic PCR from UAS-7xHalo7-CAAX flies (BL-67621). The nls and LifeAct sequences were obtained from plasmids pNLS-iRFP670 (Addgene #45466) and mCherry-Lifeact-7 (Addgene #54491). Primers and molecular details can be provided upon request.

The pUbi-FRT-nlsHalo transgene was integrated at the PB{y+,attP}VK05 (75A10) and PB{y+,attP}VK205 (99F8) sites. Similar levels of nlsHalo expression were observed upon recombination with both lines. The pUbi-FRT-LifeActHalo transgene was integrated at the PB{y+,attP}VK27 (89E) site. Injections were performed by BestGene Inc.

The following stocks were used for FLP-based recombination and nuclear labelling P{hs-FLPD5}attP40 (BL-55814), P{hsFLP}1 (BL-6), P{w[+mC] = His2Av-mRFP1}III.1 (BL-34498). Crosses were kept at 18°C to reduce leaky expression of the hs-FLP transgene. Mild heat-shocks (10–25 min at 32–34°C) were performed using a water bath. To generate a line expressing constitutively nlsHalo, the FRT-stop cassette was excised using a FLP expressed in the male germ-line (P{betaTub85D-FLP}1, BL-7196).

## Culturing of eye imaging discs, HaloTag labelling and imaging

Third instar larvae were briefly washed in water, rinsed in PBS 1x and dissected in Grace’s medium (Sigma G9771) at pH 6.7 supplemented with 5% Foetal Bovine Serum (FBS), Penicillin/Streptomycin 0.5% (Sigma P4333) and 20 nM 20-Hydroxyecdysone (Sigma H5142) [18,19]. Dissected eye imaginal discs still attached to mouth hooks were transferred to a magnetic imaging chamber (Chamlide, LCI) with a drop of culture medium. Discs were then embedded using a fibrinogen (Sigma 11,424,246)-thrombin (Sigma, 11,407,522) mix, then incubated in dissection medium supplemented with Insulin (Sigma I9278; 1.1 mg/ml). For HaloTag labelling, 1.25 μl of Janelia Fluor 646 HaloTag Ligand (200 μM in DMSO; Promega GA1120) was immediately added to the 2.5 ml of imaging medium in the imaging chamber. At room temperature and in our imaging conditions, labelling was detectable ~45 min after HaloTag addition.

Movies of nlsHalo were acquired using a Leica DMRXA microscope equipped with a 40x (PL APO, N.A. 1.32 DIC M27) objective, a Yokogawa CSU-X1 spinning disk, a sCMOS Photometrics PRIM95B camera, 491/561/642 lasers and the Metamorph software. For cell tracking in eye imaging disks, the HaloTag and RFP signals were acquired every 3.3 mins on a z-stack of typically (27 z-sections (40 mm total). Image acquisition was done at room temperature (20°C) and we did not control nor monitor the temperature of the stage during the experiments. Nuclear segmentation was performed as described earlier [20] and nuclear tracking was performed using Mastodon, an open-source framework for large-scale tracking deployed in Fiji (https://github.com/mastodon-sc/mastodon).

Confocal z-stack images of LifeActHalo were acquired from cultured explants using a Zeiss LSM780 confocal microscope (acquisition software: ZEN) with a 63× Plan Apochromat 1.4 NA differential interference contrast M27 objective.
